# Ultrasound performance using the EU-TIRADS score in the diagnosis of thyroid cancer in Congolese hospitals

**DOI:** 10.1038/s41598-022-22954-y

**Published:** 2022-11-02

**Authors:** John Bukasa-Kakamba, Pascal Bayauli, Nadia Sabbah, Joseph Bidingija, Ali Atoot, Branly Mbunga, Aliocha Nkodila, Adam Atoot, Ayrton Ilolo Bangolo, Jean Rene M’Buyamba-Kabangu

**Affiliations:** 1 Department of Endocrinology, Metabolism and Nuclear Medicine, Kinshasa University Clinics, Kinshasa, Democratic Republic of the Congo; 2 Department of Endocrinology, Metabolism and Nutrition, André Rosemon Hospital Center, University of Cayenne, Cayenne, French Guiana; 3 Department of Endocrinology, Liege University Hospital Center, Liège, Belgium; 4 School of Public Health, University of Kinshasa, Kinshasa, Democratic Republic of the Congo; 5 Department of Family Medicine, Protestant University of Congo, Kinshasa, Democratic Republic of the Congo; 6 Department of Internal Medicine, Hackensack University Medical Center/Palisades Medical Center, North Bergen, NJ USA; 7 Department of Anesthesia, Hackensack University Medical Center, Hackensack, NJ USA; 8 Antilles-French Guiana Clinical Investigation Center, Clinical Research Center (CIC), French National Institute of Health and Medical Research (INSERM) 1424, Cayenne Hospital Center, 97306 Cayenne, French Guiana

**Keywords:** Cancer, Endocrinology

## Abstract

The thyroid imaging reporting and data systems by the European Thyroid Association (EU-TIRADS) has been widely used in malignancy risk stratification of thyroid nodules. However, there is a paucity of data in developing countries, especially in Africa, to validate the use of this scoring system. The aim of the study was to assess the diagnostic value of the EU-TIRADS score in Congolese hospitals, using pathological examination after surgery as the gold standard in Congolese hospitals. This retrospective and analytical study examined clinical, ultrasound and pathological data of 549 patients aged 45 ± 14 years, including 468 females (85.2%), operated for thyroid nodule between January 2005 and January 2019. In the present study, only the highest graded nodule according to the EU-TIRADS score in each patient was taken into account for the statistical analyses. So 549 nodules were considered. Nodules classified EU-TIRADS 2 and 3 on the one hand, and, on the other hand, 4 and 5, were considered respectively at low and high risk of malignancy. The sensitivity and specificity of the EU-TIRADS score were calculated. The significance level was set at 5%. Of all patients, 21.7% had malignant nodules. They made 48.4% of the nodules in patients younger than and at 20 years old, and 31.1% in those aged 60 or over. Malignant nodules were more frequent in men than in women (30.9% vs. 20.1%; p = 0.024). Papillary carcinoma (67.2%) and follicular carcinoma (21.8%) were the main types. The malignancy rate was 39.7% and 1.5% among nodules rated EU-TIRADS 4 and 5, and those with EU-TIRADS score 2 and 3, respectively (*p* < 0.001). The EU-TIRADS score had a sensitivity of 96.6% and a specificity of 59.3%. The ROC curve indicated an area under the curve of 0.862. In a low-income country, a well performed thyroid ultrasound, using the EU-TIRADS score, could be an important tool in the selection of thyroid nodules suspected of malignancy and requiring histopathological examination in the Congolese hospital setting.

Trial registration: The research protocol had obtained the favorable opinion of the DRC national health ethics committee no. 197/CNES/BN/PMMF/2020. The data was collected and analyzed anonymously.

## Introduction

Nodular thyroid pathology is very common worldwide. Indeed, in countries where iodine intake is sufficient, the clinical prevalence of thyroid nodules is around 5% in women and 1% in men^1–5^. In the case of systematic ultrasound screening, this prevalence rises to 30 to 40% depending on age^6,7^. Ultrasound is a non-invasive diagnostic reference tool for the management of thyroid nodules; it makes it possible to assess the risk of malignancy with a validated score, the EU-TIRADS score. It allows to classify the nodules from 1 to 5 with an increasing risk of malignancy, and to select the nodules requiring Fine needle aspiration (FNA)^8–12^.

FNA is a complementary technique to ultrasound in the evaluation of thyroid nodules and allows their cytological classification according to the Bethesda score^13^. The Bethesda score makes it possible to predict the risk of malignancy with variable sensitivity (from 65 to 98%) and specificity (72 to 96%) depending on the studies^14,15^.

To the best of our knowledge, most reports on thyroid ultrasound and FNA come from developed countries. Data from developing countries are scarce and no validation study of the EU-TIRADS score has been carried out in the Democratic Republic of Congo (DRC). In addition, FNA is not readily available in medical practice in the DRC, partly due to the lack of trained specialists and a lack of pathology laboratories. The country, with an estimated population of over 80 million inhabitants, has only 5 pathology laboratories in 5 out of the 26 provinces.

The aim of the study was to assess the diagnostic value of the EU-TIRADS score in Congolese hospitals, using pathological examination after surgery as the gold standard in Congolese hospitals.

We hypothesize that the EU-TIRADS score is a good tool for selecting thyroid nodules suspected of malignancy, which must be biopsied for pathological examination in Congolese hospitals.

## Methods

This is a retrospective analytical study which included 549 patients operated on for thyroid nodules between January 2005 and January 2019. The patients were followed in 35 hospitals in the city of Kinshasa, two hospitals of the city of Bukavu and one hospital in Lubumbashi. Only files that contained the parameters of interest (clinical data, results of thyroid ultrasound and histological examination of the surgical specimen) had been included. The patients operated on for thyroid nodules, whose files missed such information, and those with incomplete or unusable results were not included. The histopathological examination was carried out in the pathology laboratories of the university clinics of Kinshasa and Lubumbashi, the National Institute of Biomedical Research, LEBOMA, the Provincial Hospital of Bukavu and the Panzi Hospital.

Using a data collection sheet, the investigators trained and enlightened on the objectives of the study, collected in the medical files, the clinical data relating to age, sex, family and personal history of thyroid disease, gravidity, parity, means of detection of the thyroid mass, duration of evolution of thyroid mass, and thyroid function.

The thyroid ultrasound reports were taken into account. The ultrasound characteristics (the overall volume of the gland, the number and size of the nodules, the solid or liquid nature of the mass, the echo structure, the echogenicity, the presence of calcification and/or satellite cervical adenopathy, the nodule vascularization) have been used by thyroid ultrasound specialists to calculate the EU-TIRADS score according to Russ et al.^16^. In the present study, only the highest graded nodule according to the EU-TIRADS score in each patient was taken into account for the statistical analyses. So 549 nodules were considered.

This score varies on a scale of 1 to 5. Only data from patients with an EU-TIRADS score greater than or equal to 2 were used for the statistical analyses, based on the assumption that patients with EU-TIRADS score of 1 corresponded to a normal ultrasound examination^16^. An EU-TIRADS score of 2 and 3 suggested nodules at low risk of malignancy and that of 4 and 5 corresponded to intermediate and high-risk nodules.

### Statistical analyses

Statistical analyzes were performed using the Statistical Package for the Social Sciences (SPSS) for Windows software version 24 and XLStat 9.2. Data are expressed as mean + /− standard deviation for metric parameters, and as absolute or relative frequencies in percentages for categorical parameters. Student's t test was used to compare means, chi-square to compare frequencies. The sensitivity, specificity, positive and negative predictive values of the EU-TIRADS score were calculated using the result of pathological examination after surgery as the gold standard. The Youden index was calculated by the following formula: Se + Sp-1, the positive (RV +) and negative (RV-) likelihood ratio respectively by the following formulas: RV +  = Se/(1-Sp) and RV − = (1-Se)/Sp. The ultrasound performance evaluation was made using the ROC curve. The comparison between the EU-TIRADS score and the pathology results was carried out using the univariate model. The threshold of statistical significance corresponded to p < 0.05.

The research protocol was approved by the DRC national health ethics committee no. 197/CNES/BN/PMMF/2020. The data was collected and analyzed anonymously, thus the need for informed consent was waived by the DRC national health ethics committee. The study was performed in accordance with the Declaration of Helsinki.

### Ethics approval and consent to participate

The research protocol had obtained the favorable opinion of the DRC national health ethics committee no. 197/CNES/BN/PMMF/2020. The data was collected and analyzed anonymously. The study was performed in accordance with the Declaration of Helsinki.

## Results

### Clinical and ultrasound characteristics of patients operated on

Table 1 represents the clinical and ultrasound characteristics for all 549 patients operated on for thyroid nodules, including 468 females (85.2%), and according to the nature of the mass. The average age of the participants was 45 ± 14 years and did not differ significantly between patients with malignant nodules and those with benign nodules. Of all patients, 21.7% had malignant nodules and 78.3% had only benign nodules. The frequency of thyroid cancer was 48.4% in patients younger than and at 20 years old, 31.1% in those aged 60 and over and 19.6% in those aged 40 to 59 years (*p* < 0.001). Compared to women, men were more affected by thyroid cancer (30.9% vs. 20.1%; *p* = 0.024). The frequency of thyroid cancer was higher among solid masses (28.6%), hypoechoic (39.7%), macronodules (32.7%), in the presence of calcification (59.4%) and satellite adenopathy (63.5%) (*p* < 0.001).

**Table 1:** Clinical and ultrasound characteristics for all patients and according to the nature of the mass.

Variables	All n = 549	Benign nodules n = 430 (78,3%)	Malignant nodules n = 119 (21,7%)	p
Age	44,5 ± 14,1	44,2 ± 13	45,3 ± 16,7	0,454
**Age range**				< 0,001
≤ 20 y.o	31(5,6)	16(3,7)	15(12,6)	
21–39 y.o	178(32,4)	151(35,1)	27(22,7)	
40–59 y.o	250(45,5)	201(46,7)	49(41,2)	
≥ 60 y.o	90(16,4)	62(14,4)	28(23,5)	
**Gender**				**0,024**
Male	81(14,8)	56(13,0)	25(21,0)	
Female	468(85,2)	374(87,0)	94(79,0)	
Duration of mass£, years	4,0(3,0–6,0)	4,0(3,0–6,0)	5,0(3,0–6,0)	0,153
Total volume£, ml	64,0(51,0–81,0)	63,0(51,0–80,0)	68,0(53,5–83,3)	0,966
**Echostructure**				** < 0,001**
Liquid	136(24,8)	135(31,4)	1(0,8)	
Solid	413(75,2)	295(68,6)	118(99,2)	
**Hypoechogenicity**				** < 0,001**
Present	259(47,2)	255(59,3)	4(3,4)	
Absent	290(52,8)	175(40,7)	115(96,6)	
**Dimension**				** < 0,001**
Micronodule	231(42,1)	216(50,2)	15(12,6)	
Macronodule	318(57,9)	214(49,8)	104(87,4)	
**Calcification**				** < 0,001**
No	480(87,4)	402(93,5)	78(65,5)	
Yes	69(12,6)	28(6,5)	41(34,5)	
**Adenopathy**				** < 0,001**
No	464(84,5)	399(92,8)	65(54,6)	
Yes	85(15,5)	31(7,2)	54(45,4)	

Values ​​are mean ± standard deviation or absolute value and percentage in parentheses. £ = the median and the 95% confidence interval. Ml = mililiter.

Significant values are in [bold].

### Types of thyroid cancer

Table 2 shows the different types of thyroid cancer. Papillary carcinoma (67.2%) and follicular carcinoma (21.8%) were the most common.

**Table 2:** Histological types of malignant tumors.

Types of cancer	Number (n = 119)	n = 119 (%)
Papillary carcinoma	80	80 (67.2)
Follicular carcinoma	26	26 (21.8)
Anaplastic carcinoma	9	9 (7.6)
Lymphoma	3	3 (2.5)
Medullary carcinoma	1	1(0.8)

### Classification of thyroid masses according to the EU-TIRADS score

**Figure 1:**
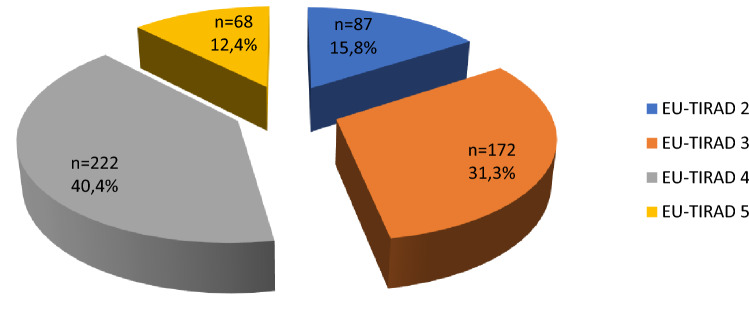
Classification of nodules according to the EU-TIRADS score.

Figure 1 shows the distribution of nodules according to the EU-TIRADS score. Nodules classified as EU-TIRADS 4 and 3 accounted for 40.4% and 31.3% respectively.

### Performance of the EU-TIRADS score in the diagnosis of thyroid cancer

**Figure 2:**
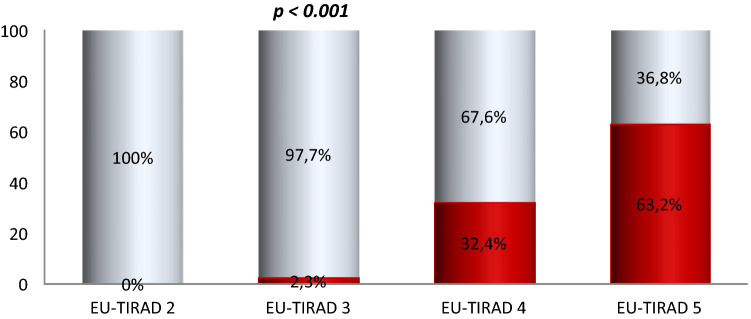
Proportion of malignant and benign nodules according to the EU-TIRADS score. Malignant nodules represented in red and benign nodules represented in blue.

Figure 2 shows the proportions of malignant and benign nodules according to the EU-TIRADS score. The proportion of malignant nodules increased significantly with the EU-TIRADS score, rising from 0 to 63.2% between EU-TIRADS scores 2 and 5 (*p* < 0.001). Out of 259 nodules classified EU-TIRADS 2 and 3, 4 (1.5%) were malignant against 115 (39.7%) among 290 with EU-TIRADS score 4 and 5 (*p* < 0.001).

Table 3 summarizes the diagnostic value of the EU-TIRADS score. The EU-TIRADS score had a sensitivity of 96.6% and a specificity of 59.3% to predict the malignancy of a thyroid nodule. Youden's index was 0.559 with a Positive Predictive Value (PPV) of 39.7% and a Negative Predictive Value (NPV) of 98.5%.

**Table 3:** Performance of the EU-TIRADS score in the diagnosis of thyroid cancer.

Probabilities	Results	CI 95%
Sensitivity (%)	96.6	95.1–98.1
Specificity (%)	59.3	55.2–63.4
Youden index	0.559	0.478–0.64
Positive likelihood ratio	2.37	1.67–3.07
Negative likelihood ratio	0.057	0.019–0.095
PPV (%)	39.7	35.6–43.8
NPV (%)	98.5	97.5–99.5
AUC	0.862	0.830–0.895

**Figure 3:**
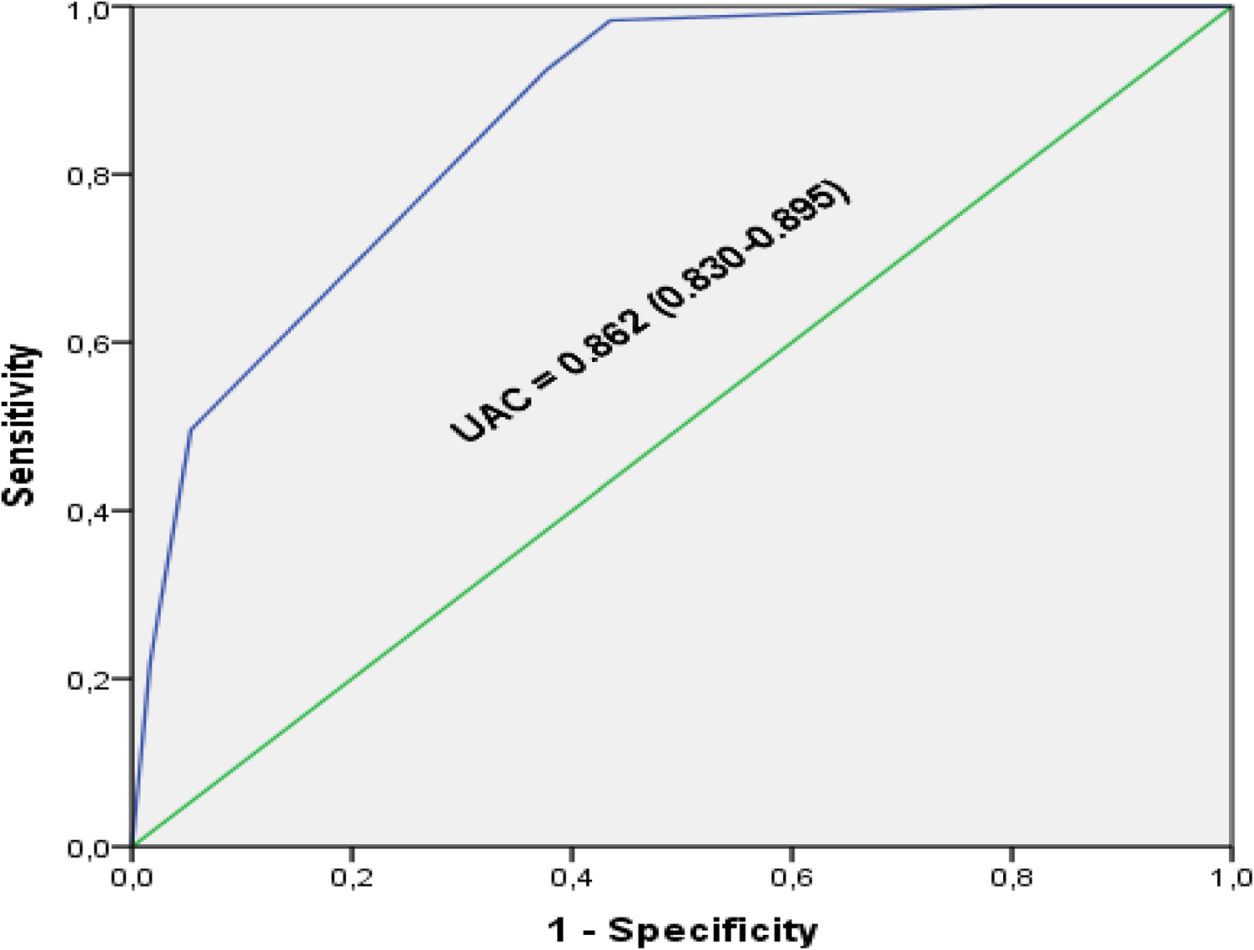
Diagnostic value of the EU-TIRADS score using the ROC curve.

Figure 3 represents the ROC curve which indicates an area under the curve of 0.862, close to 1.

## Discussion

This analytical retrospective study examined the performance of the EU-TIRADS score in the selection of thyroid nodules suspected of malignancy. The salient results indicate that 21.7% of patients had malignant nodules; the frequency of thyroid cancer was higher in patients younger than or at 20 years of age and those over 60 years of age. Compared to women, men were more affected by thyroid cancer (30.9% vs. 20.1%; *p* = 0.024). Papillary carcinoma and follicular carcinoma were the most common types and thyroid cancer was found in 1.5% of patients with nodules rated EU-TIRADS 2 or 3, and in 39.7% of those with EU-TIRADS score 4 or 5 (*p* < 0.001).

The EU-TIRADS score had high sensitivity and significant specificity in predicting malignancy. In the present study, the mean age of the patients was 44.3 ± 14.3 years. This age is close to that reported by other authors in their series^17,18^, but is lower than 54.2 years found by Léa Demasquet et al.^19^. The female predominance (85.2%) observed in our series is confirmed by the results of other authors^17–21^. The discovery of thyroid nodules was often fortuitous in our series. The ultrasound discovery of a thyroid nodule may be fortuitous in 15% of cases in the literature^22^. In the present study, of all patients, 21.7% had malignant nodules and 78.3% had only benign nodules.

The rate of benign nodules observed is in the range of 39% and 83.8% mentioned by other authors in anatomopathology laboratories^23,24^. The frequency of malignant nodules was higher in patients whose age was less than or equal to 20 years and in those at least 60 years old. The pediatric population and the elderly are at increased risk of developing thyroid cancer^25^. The reason for a high rate of cancer among patients under or 20 years old is not certain. The fact that surgery was the major criterion for the patients’ selection could be partly invoked. Surgery was the second therapeutic option after failure of medical treatment the poor response to which might have contributed to selecting suspected cases of cancer. Moreover, the glaring lack of endocrinologists justifying inappropriate care and late consultation of the doctor by patients could also be contributive.

Compared to women, men were more affected by thyroid cancer (30.9% vs. 20.1%; *p* = 0.024). The male gender has also been reported in the literature as one of the risk factors for thyroid cancer^25^. According to the literature, papillary carcinoma (67.2%) and follicular carcinoma (21.8%) are the main types of thyroid cancers^26^, similar results were seen in our study.

The distribution of benign and malignant nodules according to the EU-TIRADS score observed in our study is similar, in some respects, to that of the study by Russ et al.^16^.

Thyroid cancer was found in 1.5% of patients with EU-TIRADS 2 and 3, and in 39.7% of those with EU-TIRADS 4 and 5 (*p* < 0.001). In other words, 4 out of 10 nodules with EU-TIRADS 4 and 5 were malignant, while 0.2 out of 10 nodules with EU-TIRADS 2 and 3 showed signs of malignancy. In agreement with the literature, EU-TIRADS scores 2 and 3 corresponded to a low risk of malignancy while scores 4 and 5 reflected a high risk of malignancy^16^. Indeed, nodules classified as EU-TIRADS 4 or 5 must benefit from FNA to exclude thyroid cancer.

The sensitivity and specificity of the EU-TIRADS score observed in our study are in accordance with the literature^16,27^. Compared to the study by Léa Demasquet^19^, which reported a specificity of 31%, a 90.5% sensitivity and 46.8% diagnostic accuracy of the EU-TIRADS score, the results of our study show improved values ​​for sensitivity and specificity. Based on the results of our study, we can safely assume that thyroid ultrasound is the examination of choice in the selection of thyroid nodules suspected of malignancy, in accordance with the literature^7,28–30^. Many studies have focused on the diagnostic value of thyroid ultrasound in detecting thyroid cancer^31–35^. Several studies have also proven the importance of the EU-TIRADS score in the characterization of thyroid nodules^12,36,37^. The present study carried out in the DRC supports their results. Our study also suggests that good training in thyroid ultrasound and an improvement in the access rate of patients with thyroid nodules, to this non-invasive imaging technique, would contribute to improving the management of thyroid pathology in the country.

Certain limitations must be considered when interpreting the results of this study. Some clinical information were missing from medical files when collecting the data. Thyroid ultrasounds were performed by several doctors specializing in medical imaging, with different brands of ultrasound machines. No uniform protocol for carrying out the examination had been imposed. The bias related to the interpretation of the images by the operator and to the obsolescence of the device used could influence the quality of the data. FNA results were not available as this practice was not common during the period of this study. We do not know to what extent the results of this study can be extrapolated to all Congolese patients with a thyroid nodule in the hospital setting. However, this study has the merit of addressing the subject for the first time in the country. The sample size strengthens the results.

In a low-income country, a well performed thyroid ultrasound, using the EU-TIRADS score, could be an important tool in the selection of thyroid nodules suspected of malignancy and requiring histopathological examination.

## Data Availability

All data generated or analyzed during this study are available from the corresponding authors.

## Abbreviations

EU-TIRADS: Thyroid imaging reporting and data systems by the European thyroid association
ROC: Receiver operating characteristics
AUC/UAC: Area under the curve
DRC: Democratic Republic of the Congo
FNA: Fine needle aspiration
SPSS: Statistical package for the social sciences
PPV: Positive predicive value
NPV: Negative predictive value
Y.O: Year old
ml: Mililiter
